# The Hospital World

**Published:** 1911-07-01

**Authors:** 


### The Hospital World.

### Sir Ernest Schiff and Mr. Peter Reid.

Sir Ernest Schiff, whose name was included in the list of Coronation Honours published by us last week, was founder of the Schiff Home for Surgical Convalescents at Cobham. The home at Parkwood, Swanley, erroneously credited to him, was founded by Mr. Peter Reid, whom Lord Lytton declares to be "the pioneer in this work." Lord Lytton, it will be remembered by our readers, is chairman of the Managing Committee of the Schiff Home.

### The Architect of the Radcliffe Infirmary, Oxford.

The Bristol and West Country papers generally have been quick to note The Hospital's announcement on June 10 of Mr. Edward Warren's appointment as architect, in consultation with Dr. Mackintosh, M.V.O., of the new buildings at the Radcliffe Eye Infirmary, Oxford. Mr. Edward Prioleau Warren, they point out, is an old Cliftonian and a brother of Dr. T. H. Warren, President of Magdalen College, Oxford. He was educated at Clifton College, which he left in 1874, and some seven years later became articled to Messrs. G. F. Bodley and T. Garner, both pupils of Sir Gilbert Scott. Mr. Warren has observed his profession in various parts of the Continent. He contributed an article on the work of Messrs. Bodley and Garner some years ago to the "Architectural Review," of Boston, Mass., U.S.A. Mr. Edward Warren has done previous work in Oxford, and, like Mr. Bodley, has also had his services called in at the sister University of Cambridge.

### The Abyssinian Representative at St. Thomas's.

We are informed that the Abyssinian Representative, Dijasmatch Kassa of Ethiopia, and suite, have paid a visit to St. Thomas's Hospital, going into the details of administration and treatment. They also visited the medical school and nurses' homes, and were interested in the teaching of the nurses in the preliminary training school and in the training of the students, making inquiries in regard to the teaching of anatomy, and examined dissections. At the conclusion of the visit Dijasmatch Kassa expressed his desire to give £100 to the hospital.

### Dr. Goodhart's Distinction.

The following letter has been sent to Sir James F. Goodhart, Bart., M.D., from the Royal Hospital for Incurables, to which he is consulting physician :—

"Dear Sir,—I have been instructed by the Board of Management of this hospital to carry out a very pleasant duty in offering you their very sincerest congratulations on the honour recently conferred upon you by His Majesty the King. The Board feel that among all the gentlemen included in the list of Coronation honours no one more richly deserved the mark of Royal and State favour and interest than yourself; many years of eminently distinguished and unostentatious service have had their public reward. I heartily assure you that everybody associated with this hospital, for which you have done so much, is extremely glad.

May I add my own personal felicitations and all good wishes.—Believe me, yours very truly, (Signed) Charles Cutting, Secretary."

We are drawing no invidious distinction when we say that this honour has fallen to a gentleman who has never allowed professional interests to weigh in the scale of his uncompromising standard of good sense and public duty.

### A Doctor's Estate.

Medical men so rarely leave estates running nearly into six figures that it is worthy of note that Mr. Richard Vooght Clampitt, L.R.C., of Merton Road, Bootle, Lancs, who died on March 20, has left estate of the gross value of £98,204, with net personalty £88,415.

### Gold Medal for a Hospital Officer.

Doctor Edoardo Grandi, who for thirty-five years has been director of the Ospedale Maggiore of Milan, has just resigned his post. The Council of the Hospital Institutes recently held a special meeting and awarded him a pension and a gold medal in recognition of his services during the long time he has been associated with the charity.

### A Small Windfall for Aberdeen Hospitals.

The hospitals and charities of Aberdeen will long remember the will of the late Miss Annie Hamilton Cruickshank, daughter of the late Professor Cruickshank, of Aberdeen University, whose munificent bequests to Aberdeen University have already been made public. She bequeathed, in addition, £100 each to the Aberdeen Royal Infirmary, the Aberdeen Hospital for Incurables, the Convalescent Hospital, Aberdeen, the Aberdeen Royal Hospital for Sick Children, and £50 to the West Aberdeen Sick Men's Friend Society.

### The Duchess of Marlborough at West Ham.

The Duchess of Marlborough lately unveiled a tablet at the Woodford Cottage Hospital which is commemorative of the reign of King Edward VII. A local committee had been formed and got together subscriptions for some much-needed additions to the hospital, which had been made and paid for, while a sum had been added to the endowment funds of the hospital. The Duchess of Marlborough then motored to the West Ham Hospital, where she was met by the members of the committee. She inspected the plans for the improvements and alterations in the children's ward and assisted the committee in the selection of tiles for tiling the walls. She then presented medals and prizes to the sisters and nurses in the new Marlborough Ward, and afterwards visited each patient.

### Note.

We have been asked to state that Mr. A. S. Wills attended the recent Conference of the British Hospitals Association as the representative of the Royal United Hospital, Bath, of which he is chairman.

---

### TO HOSPITAL SECRETARIES AND OTHERS.

We invite contributions from any of our readers, and shall be glad to pay, according to length, for items of news for the institutional section (including appointments, resignations, gifts, etc.) which we may publish. All payments for manuscripts used are made as early as possible after the beginning of each quarter.

